# Oilseed Rape, Wheat, and Barley Grain Contamination as Affected by Different Glyphosate Usage

**DOI:** 10.3390/plants12061335

**Published:** 2023-03-16

**Authors:** Gražina Kadžienė, Simona Pranaitienė, Ona Auškalnienė, Agnė Veršulienė, Skaidrė Supronienė, Renata Žvirdauskienė, Viktorija Gecaitė, Jurgita Cesevičienė, Roma Semaškienė

**Affiliations:** Institute of Agriculture, Lithuanian Research Centre for Agriculture and Forestry, Instituto al. 1, Akademija, LT-58344 Kedainiai District, Lithuania

**Keywords:** pre-harvest application, glyphosate residues, metabolite AMPA (aminomethosphonic acid), grain storage, glyphosate degradation

## Abstract

Glyphosate is one of the most widely used herbicides, but is still in the spotlight due to its controversial impact on the environment and human health. The main purpose of this study was to explore the effects of different glyphosate usages on harvested grain/seed contamination. Two field experiments of different glyphosate usage were carried out in Central Lithuania during 2015–2021. The first experiment was a pre-harvest application, with two timings, the first according to the label (14–10 days), and the other applied 4–2 days before harvest (off-label), performed in winter wheat and spring barley in 2015 and 2016. The second experiment consisted of glyphosate applications at label rate (1.44 kg ha^−1^) and double dose rate (2.88 kg ha^−1^) at two application timings (pre-emergence of crop and at pre-harvest), conducted in spring wheat and spring oilseed rape in 2019–2021. The results suggest that pre-emergence application at both dose rates did not affect the harvested spring wheat grain or spring oilseed rape seeds—no residues were found. The use of glyphosate at pre-harvest, despite the dosage and application timing, led to glyphosate’s, as well as its metabolite, aminomethosphonic acid’s, occurrence in grain/seeds, but the amounts did not reach the maximum residue levels according to Regulation (EC) No. 293/2013. The grain storage test showed that glyphosate residues remain in grain/seeds at steady concentrations for longer than one year. A one year study of glyphosate distribution within main and secondary products showed that glyphosate residues were mainly concentrated in wheat bran and oilseed rape meal, while no residues found in cold-pressed oil and wheat white flour, when glyphosate used at pre-harvest at the label rate.

## 1. Introduction

Glyphosate *N*-(phosphonomethyl)glycine is a non-selective, broad-spectrum herbicide used for the control of annual and perennial plants, mainly used in agricultural fields, as well as in orchards, forests, parks, squares, railways, etc. Glyphosate acts by inhibiting 5-enolypruvylshikimate-3-phosphate synthesis (EPSPS), which is involved in the biosynthesis of aromatic amino acids [1,2]. The active substance is systematically transported to the roots and enables the effective control of perennial plants [1]. Glyphosate is very effective, has a wide range of usage and requires a relatively low cost to produce. For this reason, it is the most widely used herbicide, both across Europe [3] and the world [4]. Glyphosate use in the U.S. dramatically increased with the advent of Roundup-ready technology, which developed glyphosate-tolerant plants [2,5]. In 2014, glyphosate was used in more than 140 countries and was included in more than 750 products [4]. In 2017, glyphosate accounted for 33% of total sales of herbicide active substances (a.s.) in European countries [3]. Glyphosate accounted for more than half of the herbicide a.s. sold in Estonia, Finland, Greece, Italy, Norway and Portugal, for 20% to 50% in Austria, Belgium, Croatia, Czech Republic, Denmark, France, Germany, Hungary, Latvia, Lithuania, Netherlands, Poland, Slovenia, Spain, Sweden, Switzerland and the UK, and for less than 20% in Turkey [3]. Due to high glyphosate usage, European consumers are particularly concerned about glyphosate residues and food safety [6]. However, naturally occurring toxicants can be found in many foods regardless of agricultural practice, including both organic and conventional agriculture, and the presence of residues does not always directly equate to harm [7]. Some authors suggest glyphosate as an environmentally friendly herbicide [8,9,10], which has no effect on other organisms, when used at recommended rates [11]. Others indicate that glyphosate leaching from the soil through the rhizosphere may adversely affect non-target plants by affecting the availability of nutrients, associated with physiological disease resistance [12,13,14].

The climate crisis, the COVID-19 pandemic, and the war in Ukraine have shed light on the growing importance of resilient, sustainable, and healthy food systems. For policy makers, it is crucial to tackle the double challenge of providing food security to a growing global population, while ensuring a smooth transition to sustainable food systems and healthier diets.

Modern agricultural practices provide farmers with tools to maximize their production. Chemical weed control helps farmers grow more food on less land by protecting crops from weeds competing for essential nutrients, water, and sunlight [15]. Oerke [16] concluded that 34% of potential crop losses were caused by weeds. Poor crop rotation and minimum tillage increases the emergence of specific weeds [17,18] that require additional attention, as well as increases the use of specific herbicides. The intensive use of herbicides of the same mode of action has led to new challenges in weed control, due to the emergence of herbicide resistance, now seen in Lithuania too [19]. Desk studies conducted in Germany, the United Kingdom, France, and Sweden on the potential effects of a glyphosate ban on agricultural productivity and farm income concluded that, in particular, no-tillage farming/conservation agriculture will face severe problems without glyphosate to control weeds and cover crops [20]. Moreover, glyphosate is an important tool, controlling an invasive plant species [21,22,23].

In agriculture, glyphosate is widely used for weeds and volunteer crop control, as well as for crop desiccation purposes at pre-harvest. Pre-harvest treatment is used for cereals, rapeseed, pulses, and other crops to reduce weed biomass and to achieve more uniform ripening and faster crop harvest [24]. The pre-harvest use of glyphosate can lead to the presence of glyphosate and its metabolite aminomethosphonic acid (AMPA) in crop products, as well as in food [25,26,27]. According to annual reports of pesticide residue levels in foods and feed in the European market, for 2020, 99.4% of the analyzed samples did not exceed the maximum residue level (MRL) for glyphosate, and 94.9% of the samples were below the MRL for overall pesticide residues [28]. Potential risks of glyphosate to human health might appear via food contamination; traces of glyphosate and AMPA residues were detected in cereals, fruits, and vegetables, as well as in foods of animal origin, suggesting that these residues can be found in different food sources, including wheat grain, flour, bread, breakfast cereals, and oilseeds [29]. Recent studies in 11 EU countries showed that 21% of 317 tested agricultural topsoils were contaminated with glyphosate, and 42% contained AMPA [30].

Several studies have reported the glyphosate and AMPA residues associated with pre-harvest application [25,26,27], but not much is known about the impact of pre-emergence application, or about the possibility of glyphosate and/or AMPA uptake from soil and contamination of the harvested grain. Additionally, there are not many data available on glyphosate and AMPA changes during grain storage and processing after the harvest [27].

The objectives of our study were to explore the effect of different glyphosate usages on harvested wheat and barley grain, and oilseed rape seed, contamination by glyphosate, and investigate whether AMPA residues exceed the MRLs. We also evaluated the changes in the residual levels of wheat grain and oilseed rape seeds during storage, and the residues’ distribution in wheat white flour and bran, as well as oilseed rape oil and meal, after processing.

## 2. Results

### 2.1. The Response of Glyphosate Application Timing and Dosage on Grain/Seed Contamination by Its Residues

According to European Commission Regulation (EU) No 293/2013, maximum glyphosate residue levels (MRLs) for wheat grain and oilseed rape seeds are 10 mg kg^−1^, and for barley grain they are 20 mg kg^−1^.

The results of two -year experiments showed that rather small amounts of glyphosate and AMPA were found in harvested winter wheat and spring barley, after glyphosate application at 14–10 (14–10 DBH) and 4–2 (4–2 DBH) days before harvest (Table 1). The amounts of glyphosate residues did not exceed the MRLs for both years, despite the timing of the applications, and averaged about 11 to 25 times lower levels compared to MRLs for barley and wheat grain, respectively. No significant effects were observed between the two application timings, neither for wheat nor for barley grain contamination with glyphosate and AMPA, and no residues were found in untreated grain.

The results of the three-year experiment clearly showed that pre-emergence application had no impact on grain contamination (Table 2).

Harvested wheat grain and oilseed rape seeds were not contaminated with glyphosate or AMPA, for both pre-emergence treatments applied with glyphosate at full and double dose rates (1N-PREEM and 2N-PREEM, respectively).

Pre-harvest application led to grain contamination by glyphosate and AMPA, but even at the double dose rate (2N-PREH), spring wheat grain and oilseed rape seeds contained 4 to 11 times lower amounts of glyphosate than the MRLs (Table 2). The amounts of glyphosate obtained in wheat and oilseed rape, and AMPA in wheat grain, significantly differ in 2N-PREH when compared to those applied at the label rate (1N-PREH). Glyphosate residues were directly influenced by the application rate, and were approximately two times higher in 2N-PREH when compared to 1N-PREH, for both wheat grain and oilseed rape seeds.

### 2.2. Glyphosate and AMPA Dynamics in Spring Wheat Grain and Oilseed Rape Seeds during Storage

**Figure 1:**
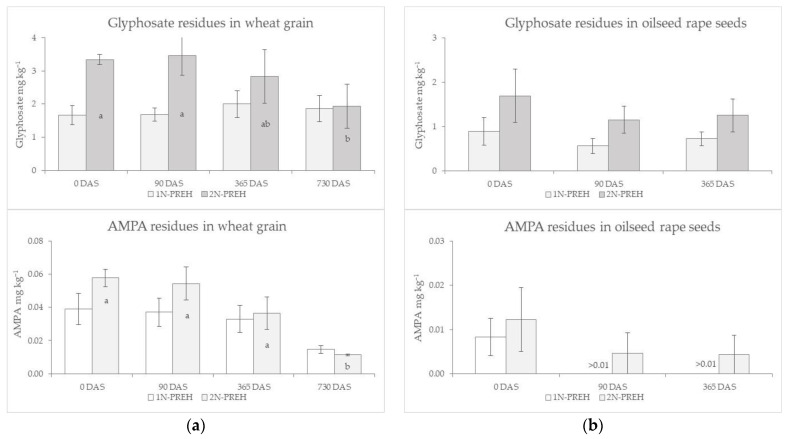
The influence of the pre-harvest application on glyphosate and AMPA residues after harvesting, at 0, 90, 365 and 730 DAS (days after storage): (**a**) in spring wheat grain; (**b**) in spring oilseed rape seeds. The bars on the columns indicate standard errors (*n*3), lowercase letters within the same type of columns mean significant differences (*p* < 0.05) for the glyphosate and AMPA residues during the storage.

Spring wheat grain and oilseed rape seed samples of 1N-PREH and 2N-PREH treatments, harvested in 2019, were examined for glyphosate and AMPA changes during storage (Figure 1). In the three months after storage, the levels of glyphosate and AMPA were almost the same as at the start of the experiment. No significant changes were obtained in the grain/seeds examined after a year of storage. Significantly lower amounts of glyphosate and AMPA residues were only found in the spring wheat grain of the 2N-PREH treatment that was stored for two years, while glyphosate levels of the 1N-PREH grain remained steady during the whole storage period.

### 2.3. Glyphosate and AMPA Distribution in Spring Wheat and Oilseed Rape Grain/Seed Production

Glyphosate- and AMPA-contaminated wheat grains and oilseed rape seeds of 1N-PREH and 2N-PREH treatments, harvested in 2021, were tested for residue distribution within the grains’/seeds’ main and secondary food/feed products, by analyzing wheat bran and white flour (Table 3), as well as oilseed rape oil and meal (Table 4). The results of a one year study showed that glyphosate residues were more concentrated on top of grain/seed layers, rather than inside, and were mainly obtained from the secondary products. Neither glyphosate nor AMPA residues were found in wheat white flour produced in the 1N-PREH-treated grain, while all the residues were found in wheat bran (Table 3). Insignificant amounts of glyphosate were obtained in wheat white flour produced in the 2N-PREH-treated grain, but the highest concentrations of glyphosate residues were found in bran.

AMPA was not detected in spring oilseed rape seeds of both 1N-PREH and 2N-PREH treatments (Table 4). Cold-pressed oil, extracted from oilseed rape seeds of the 1N-PREH and 2N-PREH treatments, was free of glyphosate residues, while all the residues were concentrated in the meal.

## 3. Discussion

Previous studies of pre-harvest glyphosate usage have reported that glyphosate application rate, crop development stage at glyphosate application time, grain moisture and possible rainfall wash plays and important role in determining the magnitude of glyphosate residues in the grain and straw [25,26]. The two-year study in western Canada showed that residues of glyphosate and AMPA in wheat grain increased with increasing rates of application, and decreased as the seed moisture content at the time of application decreased [25]. Similar results were obtained for spring oilseed rape, where higher amounts of glyphosate and AMPA residues in the seeds were associated with higher application rates, and lower amounts were associated with applications at later stages of crop development [26]. In our study, the number of glyphosate residues in wheat grain and oilseed rape seeds increased approximately two-fold, when a double dose rate applied at pre-harvest (2N-PREH) was used, compared to the normal rate—1.44 kg ha^−1^ (1N-PREH). No clear relationship between glyphosate residues and grain moisture or rainfall (between application and harvest) was obtained for oilseed rape and barley, but a slight response was found for wheat. Our study did not include application time at specific grain moistures, but only crop development from BBCH 85–87, when moisture content is usually below 30% for cereal grains and <20% for oilseed rape seeds.

**Figure 2:**
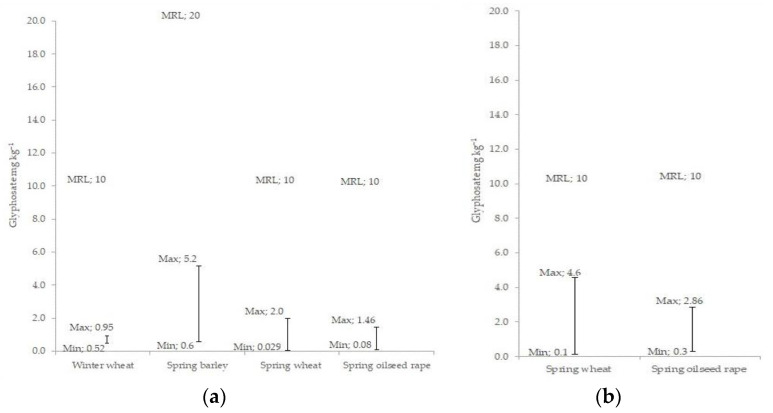
The range of glyphosate residues obtained in grains/seeds of different crops, with treatments applied at pre-harvest: (**a**) when glyphosate was applied at the label rate (1.44 kg ha^−1^); (**b**) when glyphosate was applied at the double dose rate (2.88 kg ha^−1^). MRL values indicate the maximum residue levels (mg kg^−1^) for each crop, according to Regulation (EC) No 293/2013.

Gélinas et al. have reported [31] that in wheat grain, the glyphosate residues exceeded the maximum residue level (MRL) established in Canada (5 mg kg^−1^) when the glyphosate was applied at 0.82 kg ha^−1^. The results obtained by Cesna et al. [25] showed that glyphosate residues had a concentration of <5 mg kg^−1^ in harvested wheat grain, applied at maximum rate of 1.7 kg ha^−1^, when grain moisture contents were ≤40%. In our study, concentrations of glyphosate residues in wheat grain ranged from 0.029 to 2 mg kg^−1^, when applied at the maximum label rate of 1.44 kg ha^−1^, and from 0.1 to 4.6 mg kg^−1^, when the double dose rate of 2.88 kg ha^−1^ was used (Figure 2).

Many authors indicated the lack of studies on detecting glyphosate residues in cereals, especially wheat grain [31], as well as studies comparing then with the current MRLs [27]. In our experiments, within 5 years, all the tested wheat and barley grain and oilseed rape seed samples contained lower amounts of glyphosate residues than MRLs established by the European Commission (Figure 2). The results presented by the European Food Safety Authority [28] also showed that glyphosate residues in most of the tested food and feed samples in 2020 did not exceed the MRLs. However, 283 samples (2%) were quantified at levels above the limit of quantification (LOQ), and 82 (0.6%) of 14,125 analyzed samples exceeded the MRL.

There are insufficient results on glyphosate residue in grain and its degradation due to storage [27]. Our data of grain storage showed, that after a year of storage, glyphosate residues in spring wheat grain and oilseed rape seeds were obtained at similar amounts, compared to those after the harvest (Figure 1). Approximately two times lower amounts of glyphosate and AMPA residues were only found in spring wheat grain applied at pre-harvest at a double dose rate, when stored for two years, while glyphosate levels of grain applied at pre-harvest at the label rate (1.44 kg ha^−1^) remained steady during the 2 year storage period.

A one year study of the residue distribution within whole grain/seeds and its products showed that glyphosate residues were mainly concentrated in secondary products, such as wheat bran and oilseed rape meal, rather than in white flour and oil. Granby et al. [32] also reported that glyphosate in wheat grain is more concentrated in the bran fraction after processing, while it is significantly reduced in white flour. Gélinas et al. [31] reported that no degradation of glyphosate was seen after dough fermentation and baking.

Since oilseed rape is used to produce an edible oil, glyphosate and AMPA residues in the seeds are of concern. The results of glyphosate distribution during oilseed rape processing, obtained in a one year study, look promising. The data showed that glyphosate residues were concentrated in oilseed rape meal, and no residues were found in cold-pressed oil. However, further research is needed to better understand the effect of processing on glyphosate residues in grains/seeds and their products, with respect to the end users [27,33].

Recent studies showed that in pesticide-intensive farms, various pesticides or their degradation products were found in the soils [30,34]. Silva et al. [30,34] conducted studies in 11 EU countries, where 76 residues of different pesticides or their degradation products were investigated, in 317 soils of different land use undergoing intensive pesticide usage. Studies have shown that 83% of them contain one or more residues of different pesticides and their degradation products; a total of 58% have 2 to 10 components, and 21% and 42% contain glyphosate AMPA residues, respectively. Such studies show that the intensive use of pesticides can pose a risk of environmental pollution, and this may contaminate the crop production.

Blackshaw and Harker [35] reported that even with high-dose glyphosate applications over several years, the likelihood of wheat, field pea, and oilseed rape contamination from soil residues was low. Our data suggest that pre-emergence glyphosate application was safe to use, in order prevent the contamination of spring wheat grain or oilseed rape seeds. Neither glyphosate nor AMPA were found in any spring wheat or spring oilseed rape sample (Table 2). This means that no glyphosate or AMPA from soil was transported to the grain. Nevertheless, the monitoring of glyphosate and AMPA residues in crop management systems undergoing the long-term use of glyphosate is worthwhile [35].

## 4. Materials and Methods

### 4.1. Study Site Description

The influence of glyphosate usage on grain contamination by the residues of glyphosate and one of the main degradation products—AMPA—was investigated in Dotnuva, Central Lithuania, in 2015, 2016, and 2019–2021. Two field experiments were performed, with 4 replications of each treatment in a completely randomized design. The soil was classified as an *Endocalcari–Epihypogleyic Cambisol*, of a loam texture: sand 47–52%, silt 32–33%, clay 17–21%, in a 0–20 cm layer. The glyphosate-based herbicide Roundup Classic (glyphosate 360 g L^−1^) was used for treatments in both experiments.

The first experiment of three treatments, including untreated control, was carried out in winter wheat (*Triticum aestivum* L.) ‘Famulus’ and spring barley (*Hordeum vulgare* L.) ‘Ema’ in 2015 and 2016. Winter wheat was sown in 11 September 2014 and 15 September 2015, at a seed rate of 4.5 mln ha^−1^; spring barley was sown on the 20 April 2015, and on the 4 May 2016, at a seed rate of 4.2 mln ha^−1^. Glyphosate was applied at the full registered dose rate (1.44 kg ha^−1^), according to the label, 14–10 days before harvest (DBH), following the weather conditions, at BBCH 85–87 development stage, when grain moisture was below 30%; the postponed application timing (off-label) was utilized at 4–2 DBH (Table 5), when BBCH was 87–89 and grain moisture was close to the standard 14%. The crop fields of good agrotechnical condition were selected for the experiments. The gross area of each separate plot was 45 m^2^ (5 × 9 m); the size of the harvested plot was 4.1 × 9 = 36.9 m^2^.

The second experiment of glyphosate usage at full (1N) and double (2N) dose rates (1.44 kg ha^−1^ and 2.88 kg ha^−1^, respectively) at two application timings (pre-emergence of crop and pre-harvest), including untreated controls, was conducted in spring wheat (*Triticum aestivum* L.) ‘Collada’ and spring oilseed rape (*Brassica napus* L.) ‘Lagonda’, in 2019–2021 (Table 6). Spring wheat was sown on the 16 April 2019, 9 April 2020, and 21 April 2021, at a seed rate 4.5 mln ha^−1^, and spring oilseed rape was sown on the 29 April 2019, 22 April 2020, and 21 April 2021, at a seed rate of 0.7 mln ha^−1^. Fields where glyphosates have not been used for more than 5 years were selected for experiments, to ensure glyphosate-free conditions in the soil. Both crops were sown in the same field (close to each other) every year. The parameters of each sprayed plot were 2.5 × 9 m (22.5 m^2^), and those of the harvested plot were 2.05 × 9 (18.45 m^2^). To avoid background pollution, the distance between each plot and block was 2.5 m (untreated area).

The glyphosate treatments were performed using a bicycle sprayer with compressed air, at a pressure of 0.2 MPa, equipment speed of 0.9 m s^−1^, a flat fan nozzle type, low pressure, HARDI MD-02, five nozzles spaced 50 cm apart, boom length 2.5 m, at a 40 cm distance above the target; the output of diluent (water) was 200 l ha^−1^ and wind speed was below 2 m s^−1^, with dry plant foliage and no rain for at least 6 h after application. Pre-harvest application was performed at the BBCH 85–87 crop development stage, when spring wheat grain moisture was below 30% and spring oilseed rape seed moisture was below 20%. The maintenance (fertilization, plant protection) of crops was performed according to the needs.

### 4.2. Grain Sampling, Storage and Analyses

Crop harvesting has been performed with the small plot harvester ‘Wintersteiger Delta’. All treatments of each field trial were harvested at the same date. Each separate treatment plot was harvested selectively, starting from the untreated plants, in order to avoid the introduction of glyphosate-contaminated grain. Grain samples for the analysis (approximately 0.5 kg of each plot) were immediately collected after the harvest and stored at −20 °C (for a maximum of 2 months), until their delivery to the laboratory for glyphosate and AMPA analysis.

Spring wheat and spring oilseed rape yields in 2019 were also tested for glyphosate degradation during storage. Grain samples of approximately 2 kg, for each plot, were stored under standard grain storage conditions for 2 years. The additional glyphosate and AMPA analyses were performed for untreated samples, as well as samples from both pre-harvest treatments (1N-PREH and 2N-PREH), after 3 months, 1 year, and 2 years of storage.

The glyphosate distribution in the final products of spring wheat and oilseed rape was evaluated in 2021. Grain/seed samples from untreated and both pre-harvest treatments (1N-PREH and 2N-PREH) of spring wheat and oilseed rape were collected (approximately 1 kg of each treatment) for wheat flour and bran, and oilseed rape oil and meal (oil-pressing residues) preparation.

The white flour and bran of wheat were prepared with a Quadrumat Junior mill (Brabender, Duisburg, Germany), equipped with a cylindrical sifter wrapped with a 70GG sieve (mesh opening 236 µm). The milling sequence of grain samples began in the untreated samples, followed by those from the 1N-PREH and 2N-PREH treatments. Grain moisture content for flour/bran preparation was close to the standard: 14%. Flour extraction yield was approximately 65–70%.

The rapeseed oil was extracted by using a home cold oil-press machine (1500 W). The pressing temperature was controlled within 80 °C, and the oil temperature during pressing did not exceed 30 °C. Oil extraction started in the untreated samples, followed by the the 1N-PREH and 2N-PREH treatments. Seed moisture content was close to the standard: 8%. After each sample pressing, the machine was truly cleaned of seed residues.

Glyphosate and AMPA are difficult to measure in trace analysis, due to their low molecular weights, as well as their volatility, thermal lability, and good water solubility. These properties cause problems in extraction, purification, and determination [36]. Therefore, all the glyphosate and AMPA analyses were performed in a certified laboratory, Eurofins, in Germany, by an LC-MS/MS (liquid chromatography–mass spectrometry) internal method. We subcontracted this task to Eurofins Dr. Specht International GmbH, Hamburg, which is accredited to perform this test. The analyses of the first experiment (Table 5) were performed in 2 replications × 2 years (*n*4); the second experiment (Table 6) was performed in 3 replications × 3 years (*n*9), and grain storage and final products (wheat flour and bran and oilseed rape oil and meal) from the second experiment were measured in in 3 replications × one year (*n*3). Analytical limit for glyphosate and AMPA analysis for all analysed grain/seeds and product samples was 0.01 mg kg^−1^.

According to Regulation (EC) No 293/2013, the maximum pesticide residue levels (MRLs) of glyphosate, for wheat and oilseed rape seeds at 10 mg kg^−1^ and for barley at 20 mg kg^−1^, were considered.

### 4.3. Wheather Conditions

In general, meteorological conditions slightly differed between 2015, 2016 and 2019–2021, as well as when compared to long-term mean of 1924–2021 (Table 7). The air temperature of the crop-growing season of April–August was close to the normal in 2015, 2020 and 2021 (14.1, 14.1 and 13.8 °C, respectively) and increased to 1.3 and 1.8 °C above the normal in 2016 and 2019, respectively. The amount of precipitation was 64% of the normal in 2015, close to normal in other years, and it ranged between 81 and 127% of the long-term mean. In 2015, August was extremely dry and warm, where the amount of precipitation was only 8% of the normal and the average air temperature was 2.9 °C above the long-term mean. In 2016, July and August were quite wet, where the total precipitation was 158% of the normal. In 2019, no precipitation was observed in April, and air temperature was 2.9 °C higher than the long-term mean. In August, the amount of precipitation was 144% of the normal and air temperature was 1.4 °C above the normal. In 2020, the average air temperature and amount of precipitation during April–August was very close to the long-term mean. In 2021, air temperature was close to the normal during the whole crop-growing season in April–August, but the amount of precipitation was unevenly distributed. During June and July, the amount of precipitation was 49% and 28% of the normal, respectively, while in August, it was 204% of the long-term mean.

### 4.4. Statistical Analyses

Statistical analysis was performed using statistical software SAS 9.4 (SAS Institute, Cary, NC, USA). All the data of glyphosate and AMPA analyses were subjected to a one-way ANOVA with the Fisher’s Least Significant Difference (LSD) test used for the comparisons of the means. Treatment effects were considered significant when *p* < 0.05 for the least square means.

## 5. Conclusions

The presence of the glyphosate and AMPA residues in wheat and barley grain and oilseed rape seeds reflected the use of glyphosate at pre-harvest, but the residual amounts were below the maximum residue levels, according to European Commission Regulation (EU) No 293/2013.

The residues of glyphosate and AMPA were likely to remain at similar levels after a year of wheat grain and oilseed rape seed storage.

The residues were mainly concentrated in wheat bran and oilseed rape meal, then in white flour and oil.

Neither glyphosate and AMPA residues were found in spring wheat grain nor spring oilseed rape seeds, when applied at pre-emergence.

## Figures and Tables

**Table 1:** Glyphosate and AMPA residues in harvested winter wheat and spring barley grain, applied with glyphosate at maximum label rate (1.44 kg ha^−1^) at 14–10 and 4–2 DBH (days before harvest) in 2015, 2016.

Treatment	Winter Wheat Grain				Spring Barley Grain			
	Glyphosate mg kg^−1^		AMPA mg kg^−1^		Glyphosate mg kg^−1^		AMPA mg kg^−1^	
Untreated	<0.01 a	–	<0.01 a	–	<0.01 b	–	<0.01 b	–
14–10 DBH	0.373 a	±0.175	0.034 a	±0.016	2.150 a	±1.041	0.041 a	±0.012
4–2 DBH	0.410 a	±0.207	0.045 a	±0.032	1.508 ab	±0.518	0.032 a	±0.009

<0.01 = values below the analytical limit (for the statistical analysis, they were treated as 0); ± indicates the standard error, *n*4; lowercase letters mean significant differences within the treatments, *p* < 0.05.

**Table 2:** Glyphosate and AMPA residues in harvested spring wheat grain and oilseed rape seeds, applied with glyphosate at the maximum label rate of 1.44 kg ha^−1^ (1N) and double rate of 2.88 kg ha^−1^ (2N), at two times: PREEM (at pre-emergence) and PREH (14–10 days pre-harvest), in 2019–2021.

Treatment	Spring Wheat Grain				Spring Oilseed Rape Seed			
	Glyphosate mg kg^−1^		AMPA mg kg^−1^		Glyphosate mg kg^−1^		AMPA mg kg^−1^	
Untreated	<0.01 c	–	<0.01 b	–	<0.01 c	–	<0.01 b	–
1N-PREEM	<0.01 c	–	<0.01 b	–	<0.01 c	–	<0.01 b	–
2N-PREEM	<0.01 c	–	<0.01 b	–	<0.01 c	–	<0.01 b	–
1N-PREH	1.075 b	±0.272	0.027 b	±0.006	0.412 b	±0.139	0.003 ab	±0.002
2N-PREH	2.378 a	±0.613	0.120 a	±0.071	0.929 a	±0.266	0.015 a	±0.011

<0.01 = values below the analytical limit (for the statistical analysis, they were treated as 0); ± indicates the standard error, *n*9; lowercase letters mean significant differences within the treatments, *p* < 0.05.

**Table 3:** The influence of pre-harvest (PREH) application, at maximum (1N) and double (2N) label rates, on glyphosate and AMPA residues in harvested spring wheat grain, as well as in its final products, 2021.

Treatment	Glyphosate mg kg^−1^						AMPA mg kg^−1^					
	Wheat Grain		White Flour		Bran		Wheat Grain		White Flour		Bran	
1N-PREH	0.057 a	±0.020	<0.01 a	–	0.067 a	±0.009	0.013 a	±0.002	<0.01 a	–	0.017 b	±0.003
2N-PREH	0.100 a	±0.023	0.021 a	±0.013	0.140 a	±0.026	0.024 a	±0.005	<0.01 a	–	0.042 a	±0.005

<0.01 = values below the analytical limit (for the statistical analysis, they were treated as 0); ± indicates the standard error, *n*3; lowercase letters mean significant differences within the treatments, *p* < 0.05.

**Table 4:** The influence of pre-harvest (PREH) application, at maximum (1N) and double (2N) label rates, on glyphosate and AMPA residues in harvested spring oilseed rape seed, as well as in its final products, 2021.

Treatment	Glyphosate mg kg^−1^						AMPA mg kg^−1^					
	Oilseed Rape Seeds		Oil		Meal		Oilseed Rape Seeds		Oil		Meal	
1N-PREH	0.203 b	±0.023	<0.01 a	–	0.237 b	±0.007	<0.01 a	–	<0.01 a	–	<0.01 a	–
2N-PREH	0.347 a	±0.026	<0.01 a	–	0.517 a	±0.064	<0.01 a	–	<0.01 a	–	<0.01 a	–

<0.01 = values below the analytical limit (for the statistical analysis, they were treated as 0); ± indicates the standard error, *n*3; lowercase letters mean significant differences within the treatments, *p* < 0.05.

**Table 5:** Glyphosate application at pre-harvest in winter wheat and spring barley, in 2015 and 2016.

Treatment	Application Date				Days to Harvest			
	Winter Wheat		Spring Barley		Winter Wheat		Spring Barley	
	2015	2016	2015	2016	2015	2016	2015	2016
Untreated	no application							
14–10 * DBH	31 07	22 07	31 07	12 08	14	10	14	14
4–2 * DBH	10 08	30 07	10 08	24 08	4	2	4	2

* application timing at specified intervals was performed, considering weather conditions.

**Table 6:** Glyphosate application at pre-emergence and pre-harvest in spring wheat and spring oilseed rape, in 2019–2021.

Treatment	Application date						Days to harvest					
	Spring Wheat			Spring Oilseed Rape			Spring Wheat			Spring Oilseed Rape		
	2019	2020	2021	2019	2020	2021	2019	2020	2021	2019	2020	2021
Untreated	no application											
1N-PREEM *	23 04	17 04	26 04	06 05	22 04	22 04	100	119	112	100	125	116
2N-PREEM *												
1N-PREH **	22 07	03 08	04 08	26 08	13 08	04 08	10	11	12	14	12	12
2N-PREH **												

* pre-emergence application performed after sowing, before the germination of crops; ** pre-harvest application performed, considering weather conditions, 14–10 days to harvest.

**Table 7:** Meteorological conditions.

Month	Average Air Temperature (°C)					Precipitation (mm)					The Mean 1924–2021	
	2015	2016	2019	2020	2021	2015	2016	2019	2020	2021	Air Temperature (°C)	Precipitation (mm)
April	7.0	7.1	8.9	6.8	6.0	51.5	59.5	0.0	36.9	26.4	6.0	36.8
May	11.4	15.0	12.9	10.6	12.4	50.4	27.3	55.4	51.4	100.9	12.4	51.9
June	15.1	17.5	20.6	18.9	15.8	26.3	57.4	16.1	62.3	30.1	15.8	62.0
July	17.1	18.6	17.3	17.4	17.8	57.6	128.2	66.0	76.5	21.4	17.8	76.0
August	19.7	17.1	18.2	16.8	16.8	5.6	109.2	107.0	73.3	150.8	16.8	74.1
April–August	14.1	15.1	15.6	14.1	13.8	191.4	381.6	244.5	300.4	329.6	13.8	300.8

## Data Availability

Not applicable.
